# Recovery-supportive interventions for people with substance use disorders: a scoping review

**DOI:** 10.3389/fpsyt.2024.1352818

**Published:** 2024-03-21

**Authors:** Deborah L. Sinclair, Mégane Chantry, Clara De Ruysscher, Jürgen Magerman, Pablo Nicaise, Wouter Vanderplasschen

**Affiliations:** ^1^ Department of Special Needs Education, Ghent University, Ghent, Belgium; ^2^ Institute of Health and Society (IRSS), UCLouvain, Brussels, Belgium; ^3^ EQUALITY//ResearchCollective, HOGENT University of Applied Sciences and Arts, Ghent, Belgium

**Keywords:** recovery-supportive interventions, recovery-oriented, substance use, mental health disorders, scoping review

## Abstract

**Background:**

Recovery-supportive interventions and strategies for people with substance use disorders are a cornerstone of the emergent recovery paradigm. As compared to other services, such approaches have been shown to be holistically focused and improve outcomes (e.g. substance use, supportive relationships, social functioning, and well-being). Even so, a comprehensive overview of the nature, extent, and range of research on the topic is lacking.

**Methods:**

A scoping review of the literature was conducted to characterize the main topics on recovery-supportive interventions. A systematic search was conducted in three databases: Scopus, Web of Science, and PubMed from January 2000 to July 2023 using the PRISMA-ScR. Twenty-five studies published between 2005–2022 met the inclusion criteria.

**Results:**

Most studies emanated from the United States, and we found a peak in publication frequency between 2018–2022 (n = 13) relative to other years. The most prominent lines of inquiry appear to concern recovery-oriented policies; principles of recovery-oriented services (challenges encountered when implementing recovery-oriented practices, relationships with service providers characterized by trust, and service user-service provider collaboration), and recovery capital (particularly recovery-supportive networks, employment, and housing). Seventeen studies addressed co-occurring disorders, and eight addressed substance use recovery.

**Conclusion:**

To advance the field, more context-specific studies are required on supporting peer professionals, (including enabling cooperation with service users, and hiring experts by experience as staff), and training of professionals (e.g., nurses, psychologists, social workers, physicians) in the principles of recovery.

## 1 Introduction

A central concern of professionals who work within the substance use treatment arena has been the development of effective strategies and interventions to promote recovery. Addiction recovery has been defined as “a voluntarily maintained lifestyle characterized by sobriety, personal health, and citizenship” (1) (p. 222) and is the goal of services and an organizing framework (2). As a goal, recovery transcends abstinence to encompass a purposeful, self-determined life (3). As an organizing concept, the recovery paradigm stands in contrast to the preceding pathology-oriented and treatment-focused paradigms, putting forward that the principles and practices that can support stable recovery can be derived from the lived experiences of individuals in recovery, their families and communities to benefit others’ recovery initiation and maintenance efforts (4).

Research indicates that, as compared to other services, recovery-supportive interventions and strategies (from hereon recovery-supportive interventions) explicitly value the inclusion of experts by experience, prioritize independence, self-determination, empowerment, and regard for service users to yield improved outcomes (e.g. substance use, supportive relationships, social functioning, and well-being) (5–7). A recovery orientation suggests the central involvement of people in recovery, the community, and service and support providers (8) while recovery-supportive interventions encompass a broad range of actions that directly or sequentially facilitate change through various mechanisms (9). The change toward recovery-supportive interventions necessitates the preparation of the mental health and addictions workforce with recovery-based clinical skills and tools, mechanisms, and structures (10, 11). However, while the knowledge base on recovery-supportive interventions continues to expand, there exists a gap between recommendations and practice (12).

As recovery-supportive interventions operate within complex systems, determining the scope of the related literature is a much-needed step toward encouraging greater adoption and offering practice recommendations to address barriers to recovery. While researchers have recently sought to synthesize research on recovery-supportive interventions for individuals with substance use disorder (13), the scope was limited in terms of disciplinary focus (nursing), time range (2010–2019), and review methodology (narrative review).

Consequently, we sought to synthesize the available literature on recovery-supportive interventions for adults who use substances using a scoping review methodology. Scoping reviews offer an overview of a particular area, examining the extent, nature, and range of research activity and summarizing and disseminating research (14). Exploring extant literature has important implications for re-envisioning existing care systems and promoting the transformation toward recovery-focused practice.

## 2 Methods

Arksey and O’Malley’s (14) methodological framework guided this scoping review and entailed: (a) forming a research question; (b) retrieving relevant literature; (c) selecting literature; (d) data extraction, and (e) synthesizing and outlining the results. No review protocol was registered or published for this study. When reporting on the review the Preferred Reporting Items for Systematic Reviews and Meta-Analyses extension for Scoping Reviews (PRISMA-ScR) checklist was followed (15).

### 2.1 Step 1: Developing a research question

The research question guiding this review was: what is the scope of the available literature on recovery-supportive interventions for people with substance use disorders?

### 2.2 Step 2: Identifying relevant literature

The databases were selected in consultation with the literature. We conducted a preliminary search to identify search terms and subsequently searched Scopus, Web of Science, and PubMed for English-language articles published between January 2000 and July 2023. We repeated our search in October 2023. No restrictions were placed on the study design. The two sets of search terms used were “recovery-oriented intervention”, “recovery-oriented approach”, “recovery-oriented practice”, “recovery-oriented care”, “recovery-oriented service”, “recovery-oriented model”, “recovery-supportive” and “substance use”, “substance misuse”, “substance abuse”, “substance dependence”, “substance use disorder”. The included studies were reference mined to identify additional pertinent studies.

### 2.3 Step 3: Selecting literature

**Figure 1:**
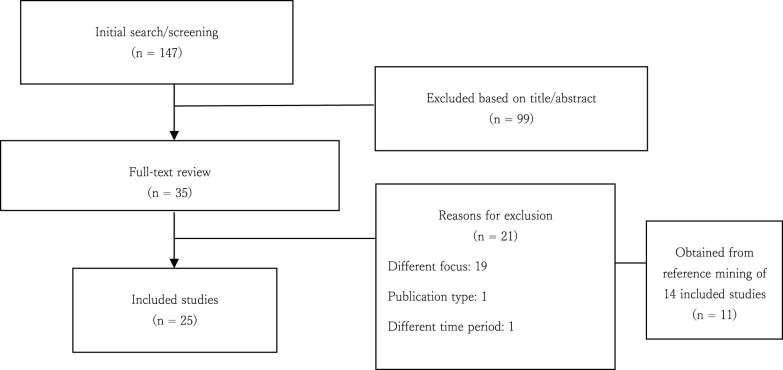
Flowchart describing the study selection process.

We focused on publications that reported on recovery-supportive interventions for persons who use substances and persons with co-occurring mental health and substance use disorders. Only adult samples (aged 18 and older) were eligible. We included only scientific research articles; books, chapters, editorials, conference presentations, commentaries, literature reviews, and grey literature were excluded. The study selection process entailed screening titles and abstracts and reviewing full texts. The initial search yielded 147 potential publications across all databases (PubMed = 45; Scopus = 49, and Web of Science = 53). Following title and abstract screening for relevance, 48 studies remained from which 13 duplicates were removed (n = 35). A further 11 articles were identified through reference mining (n = 46). In all, 122 articles were excluded and 25 publications were retained for review. **Figure 1** depicts the study selection process.

### 2.4 Step 4: Charting the data

The data extraction categories were: country, study aim, focus (substance use or co-occurring disorders), method, and key findings. Data were synthesized using textual narrative synthesis (16).

### 2.5 Step 5: Presenting the synthesized results

We identified 25 articles, published between 2005 and 2022. The key attributes of these articles are presented in **Table 1**.

**Table 1:** Overview of the included studies.

Country	Authors	Study aim	Substance use (SU) or co-occurring disorders (COD)	Data sources	Key findings
Belgium and The Netherlands	Bellaert et al. (2021) (17)	To contrast addiction recovery vision, application, and evaluation in Belgium and the Netherlands	SU	A focus group discussion, interviews, policy documents	While the recovery paradigm was openly declared, structural implementation, earmarked funding, and systematic assessment of policies are deficient in both settings.
USA	Bergman et al. (2015) (18)	To investigate whether participation in 12-step groups confer benefits apart from professional continuing care services	SU	Survey	Active participation in 12-step groups and recovery-supportive, professionally-directed services may improve outcomes following residential treatment.
Norway	Brekke, Lien, Nysveen & Biong (2018) (12)	To identify service provider dilemmas in delivering recovery-oriented support to people with co-occurring disorders	COD	Focus group discussions	Dilemmas related to recovery-oriented practice: “(1) balancing mastery and helplessness (2), balancing directiveness and a non-judgmental attitude, and (3) balancing total abstinence and the acceptance of substance use” (p.1). Understandings of recovery-oriented practice differed.
Norway	Brekke, Lien, Davidson & Biong (2017) (19)	To elucidate experiences of recovery among service user with co-occurring disorders	COD	Individual in-depth interviews	Major recovery barriers were housing and finance-related. The study lends support to services that integrate social services and health care and are collaborative.
Norway	Brekke, Lien & Biong (2018) (20)	To illuminate characteristics of professional helpers supporting recovery from co-occurring disorders	COD	Individual in-depth interviews	Professionals built trust with service users through “(a) hopefulness and loving concern, (b) commitment, (c) direct honesty and expectation, and (d) action and courage” (53).
Sweden	Cruce et al. (2012) (21)	To explore people with co-occurring disorders' perspectives of recovery-promoting care	COD	Individual in-depth interviews	To foster recovery, care should involve cooperation between the service provider and the service user to meet the latter’s needs and consolidate their participation.
Belgium	Dekkers et al. (2020) (22)	To uncover the recovery-supportive elements of NA	SU	Individual in-depth interviews	A non-judgmental approach and mutual understanding promoted Connectedness (e.g. building a social network).
USA	Felton et al. (2006) (23)	To examine an ACT team’s responses to recovery training	COD	Observations	Challenges in the delivery of recovery-oriented services were: reconciling the treatment system and service users’ goals, forging collaborative service user-service provider relationships, and implementing a recovery orientation amidst service user crisis and/or denial.
USA	Francis et al. (2020) (24)	To explore how women in substance use recovery manage their social networks post-treatment	SU	Computer-assisted interviews	Recovery was sustained by disconnecting or having less contact with people that can endanger recovery and connecting with recovery-supportive people.
USA	Green et al. (2015) (25)	To examine participants’ substance-related recovery experiences	COD	In-depth interviews	Flexible treatment approaches, reducing barriers to engagement, supporting psychoeducation, and adopting a chronic disease model may increase participation and positive outcomes, while peer support groups can help people with serious mental illness.
USA and UK	Humphreys & Lembke (2014) (26)	To scrutinize recovery-oriented policy in the USA and UK	SU	Public policy	Available rigorous research supports that recovery-oriented interventions (e.g. recovery housing and programs that expand peer support within formal treatment) improve individuals’ substance use and health outcomes cost-effectively.
Australia	Isaacs & Firdous (2019) (27)	To demonstrate how a care coordination model can promote interagency collaboration	COD	Theoretical/Exemplars from the literature	A care coordination model could address most challenges that impede service system integration.
Denmark	Jørgensen et al. (2022) (28)	To investigate the practical application of recovery-orientation in mental health centers	COD	Focus group discussions	Aspects considered important for recovery-oriented services are relationships, trust, interest, spending time with service users, and being hopeful.
Canada	Khoury (2019) (29)	To elucidate how recovery-oriented mental health policies are enacted in an ACT team	COD	Case studies	Egalitarian exchanges between service providers and service users enable the co-construction of innovative practices.
Norway	Kvia et al. (2021) (11)	To investigate health care services change towards a recovery-supporting model in a Norwegian municipality	COD	Focus group discussions	Three themes emerged: “reflections on attitudes and actions, patients not participating in matters regarding their situation, and balancing paternalistic attitudes and patients’ autonomy” (p. 1919), and understanding recovery but not knowing how to apply it practically.
USA	Laudet & White (2010) (30)	To document the priorities of persons in recovery to guide the development of recovery-oriented systems	SU	Computer-assisted interviews	Leading priorities were employment and education, family/social relations, and housing, while employment remained in the lead across recovery stages.
USA, England, Scotland, the Republic of Ireland, Denmark, and New Zealand	Le Boutillier et al. (2011) (31)	To identify the essential features of international recovery-oriented practice guidance and develop an overarching conceptual framework to aid its application	COD	International documents	Four practice domains emerged: fostering citizenship, commitment from the organization, supporting recovery (as it has been self-defined), and the working relationship.
France	Loubière et al. (2022) (32)	To evaluate the impact of the Housing First model among people with high support needs who are homeless	COD	A multi-center randomized controlled trial	The 4-year follow-up demonstrated higher housing stability, self-sufficiency, and less use of hospital services in the Housing First group compared to the Treatment-As-Usual group, however, problems with alcohol endured.
USA	Martin et al. (2022) (33)	To identify service user and provider-reported elements of the perinatal transition that impact recovery in women receiving medication-assisted treatment	SU	In-depth interviews	Clinical (e.g., neonatal opioid withdrawal syndrome), psychosocial, and mental health aspects, as well as stigma and mistrust from service providers, promote and challenge recovery.
Norway	Nesse et al. (2022) (34)	To explore the potential benefits of collaborative recovery-oriented practice development for supported housing residents	COD	Survey	Participants at the project site reported an increased willingness to ask for help.
USA	O’Connell et al. (2005) (35)	To assess the perceived implementation of recovery-oriented practices in mental health and addiction agencies	COD	Survey	The highest ratings were assigned to agencies for helping people explore their interests and the lowest on service user involvement in services.
Belgium	Pouille et al. (2021) (36)	To explore first-person perspectives of MEM in recovery from problem substance use; explore recovery capital and barriers	SU	In-depth interviews	Recovery capital is impacted by MEM-specific elements (culture, identity as an immigrant, experiencing stigma, and facing structural inequalities).
USA	Salyers & Tsemberis (2007) (37)	To investigate whether a recovery-oriented approach can be integrated into Assertive Community Treatment (ACT) while maintaining fidelity to the program	COD	Theoretical	Recovery-oriented ACT practices can be promoted by: incorporating other evidence-based practices; observing recovery orientation; delivering recovery-oriented training and supervision; and hiring service users as staff.
Australia	Thomas & Rickwood (2016) (38)	To explore one woman’s ongoing recovery experience	COD	In-depth interviews	Recovery-supportive services included individualized clinical support, therapeutic groups, and support towards self-management.
USA	Tsai & Rosenheck (2012) (39)	To assess a peer-support model of case management in a supported housing program for people with co-occurring disorders experiencing homelessness	COD	Administrative data	The model was linked to greater increases in social integration, receipt of more case manager services, and faster procurement of housing vouchers.

## 3 Results

### 3.1 Sample

The majority of studies were from the United States (18, 23–25, 30, 33, 35, 37, 39) and two were cross-national covering the USA and UK (26) and the USA, England, Scotland, the Republic of Ireland, Denmark, and New Zealand (31). Five studies emanated from Norway (11, 12, 19, 20, 34), two from Belgium (22, 36), and a third based on data from Belgium and the Netherlands (17). Two studies originated in Australia (27, 38), while single studies emerged from France (32), Canada (29), Denmark (28) and Sweden (21). A third of the studies (n=8) addressed substance use recovery while two-thirds (n=17) addressed co-occurring disorders.

### 3.2 Methodological features of the studies

Most studies were qualitative in design (n = 17; 68%), 5 were quantitative (20%), 2 were theoretical (8%) and 1 (4%) was a policy analysis (another qualitative study had a policy analysis component). The qualitative studies were predominantly underpinned by individual interviews as a data source (19–22, 24, 30, 33, 36, 38); three studies employed focus group discussions (11, 12, 17, 28). One study utilized both interviews and focus groups (17).

**Figure 2:**
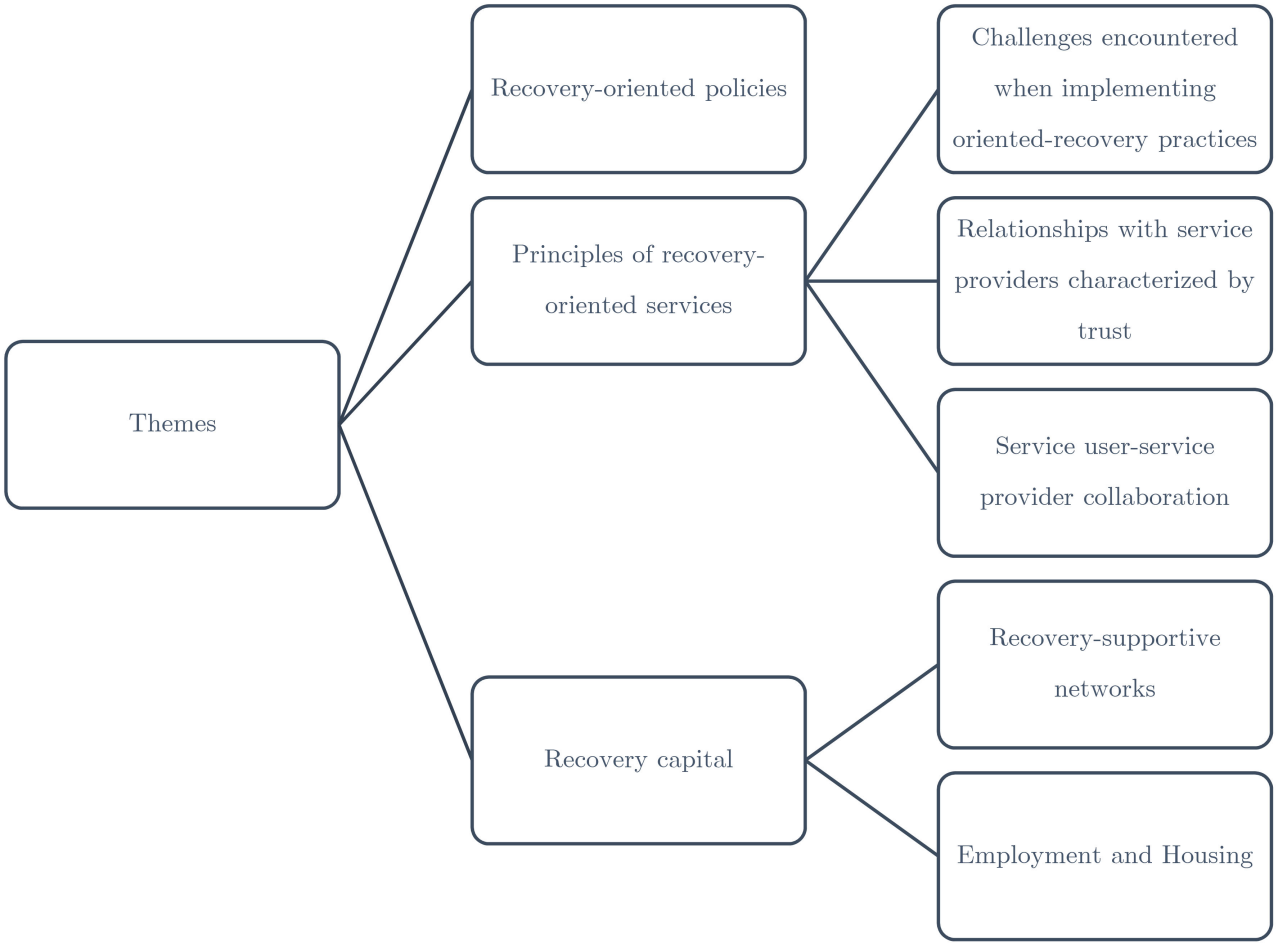
Overview of key themes.

The key themes to emerge from this scoping review pertained to recovery-oriented policy; the treatment system and service dynamics (e.g. trust, collaboration); and recovery capital (housing, employment, recovery-supportive networks). **Figure 2**

### 3.3 Recovery-oriented policies

Three studies (17, 26, 27) foregrounded the importance of recovery-oriented policies as fundamental in delivering recovery-supportive interventions. In an analysis of addiction sector policy in Flanders (Belgium) and the Netherlands, Bellaert and colleagues (17) found that beneath the rhetoric of recovery, there were deficits in structural implementation, funding allocations, and methodical evaluation of recovery-oriented policies. Thus, they advocate for the inclusion of experts by experience and the alignment of funding and policies. In a study contrasting the USA and UK’s recovery-oriented policy and care systems (26), it was revealed that the USA dedicates significant funding in support of pro-recovery treatment system transformation and towards recovery community organizations whereas, in the UK, much of the recovery-supportive interventions were yet to be evaluated. The available robust research indicated that recovery-supportive interventions (referring here to recovery housing, programs that facilitate 12-step mutual aid engagement, and the expansion of peer support within formal treatment programs) cost-effectively improve substance use and health outcomes (26). In another study, Isaacs and Firdous (27) advocated that, in the design of recovery-oriented services, a care coordination model could facilitate interagency collaboration. Their model, emanating from Australia’s Partners in Recovery initiative employed a care coordinator to serve as the point of contact between service users and service providers, resulting in a stronger therapeutic alliance and a more holistic approach.

### 3.4 Principles of recovery-oriented services

Five studies addressed aspects of the treatment system, treatment service, and/or service provider factors in the provision of recovery-supportive interventions. An analysis of recovery-oriented practice guidance from six countries identified four practice domains, namely the need to advance citizenship and reintegration into society to live as equal citizens, commitment from organizations to a conducive work environment and service structure, supporting individuals’ recovery goals, and a working relationship that demonstrates genuine support and partnership (31). A case study illustrates how a recovery orientation can develop personal responsibility within the service user for the benefit of recovery (38). One service user was interviewed during three separate admissions to a residential mental health unit. Impactful attributes of the service that were instrumental in fostering their recovery were tailored clinical support, assistance with meeting practical needs, participation in therapeutic groups, social interaction with fellow service users and staff, and support in developing self-management capacities.

#### 3.4.1 Challenges encountered when implementing oriented-recovery practices

In a Norwegian study with service providers from a mental health and substance use unit, Kvia et al. (11) concluded that although they understood the tenets of recovery, there was uncertainty about the practical steps to be taken toward transformation to a recovery-supportive model. Although participants reflected on their actions and attitudes, reflection did not extend beyond existing practice to ways in which positive changes could be made. Another prominent theme was the failure to involve service users in organizing their care. Relatedly, service providers recognized the tension between acting paternalistically and the need to support service user autonomy and empowerment. As a result, structures, tools, and mechanisms are needed for practical guidance. In a qualitative study of Norwegian service providers (12), the challenges inherent in delivering recovery-oriented care to people with co-occurring disorders were explored. Dilemmas included ‘balancing mastery and helplessness’ (the tension between helping and infringing on service users’ responsibility; guarding against disempowering service users while ensuring they do not hinder change efforts), ‘balancing directiveness and a non-judgmental attitude’ (basing treatment goals on what is important for help-seekers without judging how people live their lives, or being indifferent to their decisions; adopting a non-judgmental attitude), and ‘balancing total abstinence and the acceptance of substance use’ (adopting a professional, non-moralistic attitude, remaining supportive and hopeful amidst relapse). Attending to these dilemmas will necessitate innovative approaches to practice development. Lastly, Salyers and Tsemberis (37) offer four recommendations to establish recovery-oriented assertive community treatment (ACT) practices: integrating other evidence-based practices; monitoring recovery orientation; providing recovery-oriented work training and supervision, and hiring service users to join as staff.

#### 3.4.2 Relationships with service providers characterized by trust

Another aspect of a recovery orientation was the need for a trusting relationship between service providers and service users. Martin et al. (33) conducted interviews with nine providers from an outpatient addiction clinic and 12 women receiving treatment for an opioid use disorder to identify influential factors in the pregnancy to postpartum transition that promote or hinder recovery. Stigma and mistrust by child welfare and healthcare providers challenged recovery and provided insight into how recovery-oriented care can be promoted for families affected by opioid use disorder. Jørgensen, Hansen, and Karlsson’s (28) study with healthcare professionals rendering care to service users experiencing co-occurring disorders emphasized the need to balance forming trusting relationships, hopefulness about service users’ futures, time spent with service users, and respecting their life experiences and knowledge with their role of stabilizing health and realizing self-care. Another study on recovery from co-occurring substance use and mental health disorders explored eight peer support workers (with lived experience) behaviors and attributes (20). Trust was a cross-cutting factor in the identified themes. Trust was established and maintained by professionals when helping people with co-occurring disorders through hopefulness and loving concern (i.e. expressing their belief in a better future life which helped participants reclaim hope), commitment (ongoing, long-standing relationships with service users leading to honesty), honesty and sharing expectations (frankness and raising concern about the severity of participants’ situation, and offering guidance on change as the need for change was better understood), and action (urging participants to be more active and initially practically supporting them, enabling them to avert loneliness, and acquire confidence in their newly-acquired skills).

#### 3.4.3 Service user-service provider collaboration

Four studies highlighted the collaborative relationship between service users and service providers as underpinning recovery-oriented practices. One statewide survey of 78 mental health and addiction programs administered the novel Recovery Self Assessment measure to multiple participant groups, including agency directors, service providers, people in recovery, their families, and significant others to assess the degree to which respondents perceived recovery-oriented practices were being implemented. Although the highest-rated items related to services support of service users’ aspirations and interests beyond symptom alleviation, services were rated lowest on items concerning service user engagement in the design, management, and delivery of services (35). Another study focused on the challenge of developing more recovery-oriented practices (34) compared supported housing provision within an ongoing collaborative recovery-oriented practice development initiative (n = 7) to a reference group following practice as usual (n = 21). Findings reveal that residents at the project site exposed to the recovery-oriented practice development reported a significant increase in the recovery domain of *willingness to ask for help*. The authors contend that such a collaborative approach can support the recovery and protect residents’ citizenship in supported housing. According to Khoury (29) (p. 1), although “the (over)use of medicolegal tools and the unchanging conception of ‘madness’ represent obstacles to the sustained development of interventions centered on the person, his living conditions, and his recovery” service provider-service user interactions grounded in positive and egalitarian relations facilitate the co-construction of innovative practice approaches and signal the potential for recovery-supportive interventions. In Felton and colleagues’ US study (23), ACT team members expressed that challenging recovery-oriented tasks were the following: aligning system-centered and service-user goals, developing collaborative relationships with service users, and applying a recovery orientation during service user crisis or denial of their illness. A sample of people with co-occurring mental health and substance use disorders typified recovery-promoting care as offering empowerment and in so doing, increasing their motivation and capacity to actively engage in their recovery journey (21).

### 3.5 Recovery capital

Recovery capital refers to the personal, social, and community resources that are the basis for personal recovery and the “resources and capacities that enable growth and human flourishing” (34, p. 305-306). Sub-themes that emerged from the analysis included *recovery-supportive networks* and *employment and housing.*

#### 3.5.1 Recovery-supportive networks

Five diverse studies discussed the value of recovery-supportive networks for recovery. In a study of Narcotics Anonymous members, Connectedness [in the context of the CHIME-D personal recovery framework, Connectedness, Hope, Identity, Meaning in life, Empowerment, and Difficulties (40),] emerged as the leading recovery-supportive element of the fellowship. Connectedness was underpinned by the fellowship members’ non-judgmental approach and mutual understanding. Connectedness was central to establishing a social network (22). It has been argued that peer-based addiction recovery support (e.g. Alcoholics or Narcotics Anonymous) can be beneficial for people with mental health disorders particularly when accepting of psychiatric medications (25).

Francis et al. (24) delved into the post-treatment experiences of 88 women to reveal that, disengaging from or reducing communication with people that endanger recovery and expanding their networks to include people who support recovery was necessary for recovery maintenance. As women are said to find it especially challenging to develop recovery-supportive networks, these findings benefit service providers seeking community integration for these service users. In the only study to explore the recovery experiences of migrants and ethnic minorities (36), the development of recovery-oriented systems of care was said to be contingent on the provision of culturally competent services, efforts to ameliorate structural barriers, and, notwithstanding the many universal elements of recovery capital, the recognition that access to recovery resources are intertwined with migration status. Environments that optimize opportunities to build culturally sensitive community recovery capital, and meaningful social networks (social recovery capital) were considered essential for promoting an enduring recovery. Likewise, Bergman and co-authors (18) highlight community recovery capital in their assertion that active participation in 12-step mutual aid groups and involvement with recovery-supportive, professional services that strengthen ties to community assets potentially enhance the gains of residential treatment.

#### 3.5.2 Employment and housing

The practical need for employment and housing was identified as a key priority for recovery-oriented systems and services. Insights from 356 people at various stages of recovery demonstrate that, while housing, education, and family/social relations remain challenging long after attaining abstinence, employment remains the leading priority regardless of the recovery stage (30). Similarly, in a study on the recovery orientation of services in a district of Norway, financial difficulties (with limited potential solutions) and precarious and inadequate housing were identified as threats to recovery among people with co-occurring substance use and mental health disorders. The articulated dimensions of recovery were less tangible: cultivating self-love, feeling accepted by and useful to fellow citizens, gaining mastery over one’s life, and the emergence of the self. The findings suggest that services should be designed so as to allow for integrated health care, social services, and inter-service collaboration (19). Tsai and Rosenheck’s (39) study investigated the outcomes of a ‘group intensive peer-support model of case management for supported housing’, finding that as compared to the reference sites this form of peer support was linked to a larger increase in perceived social integration, more case management services, and faster procurement of housing vouchers.

The one randomized control trial included in this review focused on homeless individuals with mental health disorders (32). Follow-up of the sample revealed improvements in personal recovery outcomes, higher housing stability, independence, and lower use of hospital services compared to the treatment-as-usual group, but, enduring issues with alcohol (32). Findings speak to the long-term benefits of this intervention for this population.

## 4 Discussion

This scoping review has identified and analyzed 25 studies on recovery-supportive interventions published between 2005–2022. The most prominent research avenues appear to concern recovery-oriented policy; treatment services (including provider-related trust and collaboration), and recovery capital (particularly recovery-supportive networks, employment, and housing). Most studies were from the United States, and we found a peak in publication frequency in 2018–2022 (n = 13) relative to other years. Seventeen studies addressed co-occurring disorders, and eight addressed substance use recovery. The emphasis on recovery-oriented policies, their implementation, the need for systematic evaluation, intra-agency collaboration, the inclusion of experts by experience, and funding allocations (17, 26, 27) is borne out in the literature.

The included studies underscore that countries differ in their policies and practices for attending to mental health disorders and substance use, and recovery orientation. As Humphreys and McLellan (41) accentuate, “how treatment systems are structured, organized, staffed and supported fiscally varies enormously throughout the world, such that a service improvement strategy that works well in one country may be ineffectual in another” (p. 2064). This suggests that the actions needed to orient services toward recovery must be designed for the target treatment system and that service goals may best be assessed therein. For example, recent findings from the US illustrate that a relatively nominal percentage of the funding for substance use prevention and treatment is allocated to recovery (42). That said, meaningful engagement with stakeholders (including service users) has been found to be critical to positive outcomes for service users and the care system when funding changes (increases, decreases, funds being reallocated, or a different funding model applied) (43).

A recovery orientation requires that service providers approach their tasks and interactions with the service user in a particular manner. Certain practical dilemmas that have arisen for service providers include finding a balance between helping and supporting and disempowering service users, being led by service users in setting treatment and recovery goals, and adopting a professional, supportive, and hopeful attitude amidst relapse (12). While the principles of recovery were understood, translation, or how to practically approach the transformation towards a recovery orientation could be unclear (11), and practical guidance on good practice was needed (12). Our finding that collaboration and trust are two key elements in the delivery of recovery-supportive interventions is congruent with the literature (44). For some service providers it remained challenging to forge collaborative relationships with service users. The service provider has been described as “walking alongside” service users and their families when collaborating with them. Such collaboration necessitates that service providers are led by the service user concerning their recovery goals and aspirations and that a working relationship is negotiated (45). Moreover, as partnerships are collaborative, recovery-oriented professionals take on the complexities and the uniqueness of the change process. Another aspect of cooperation with service users is to hire them as staff (37). Yet, the presence of peer support workers in and of itself does not guarantee that a service operates within the recovery paradigm. Rather, the organization should be committed to respecting, supporting, promoting partnership with and delineating the role of peers (46).

Recovery-supportive interventions also centered around the development or growth of recovery capital. Recovery capital is known to accrue and deplete during ‘active addiction’ such that “most clients entering addiction treatment have never had much recovery capital or have dramatically depleted such capital by the time they seek help” (37, p. 30). In particular, housing, employment, and recovery-supportive networks were the focus of several interventions. The studies included in this review reinforce that these three areas of functioning remain a priority across recovery stages (30). Best (47) reports on the “Jobs, Friends and Houses (JFH)” project which seeks to support an enduring recovery by focusing on these same elements of recovery capital. These findings are echoed in a more recent study where stable housing, access to peer support, and care coordination were instrumental in building recovery capital, promoting recovery, and decreasing reoffending (48). A recovery capital lens holds promise for practically supporting complex populations, transcending a shortcoming-oriented approach, and steering practitioners toward the most suitable interventions (49). Therefore, we invite clinicians, care professionals, health care managers, and providers to re-center their activity towards recovery/social capital as a priority alongside medical and psychological treatment.

Concretely, our findings lend itself to the following recommendations:

• Through a collaborative, participatory process extant policies should be revised by involving persons with lived experiences (cf. 33). Policies governing the provision of services can help re-orient service towards recovery by directing the allocation of funds, reconfiguring how care is organized and by whom it is rendered, and shaping the relationships within and between services.
• Relatedly, service providers and peer workers should undergo continuous training. In the case of the various professionals employed within addiction care, their foundational training should equip them to operate from within a recovery approach. Continued professional development courses can help ensure that their skills continue to be honed and that organizations continue to build their capacity. In the case of peer workers, training programs should also be designed collaboratively and with their needs in mind. Through dialogue and openness, the training can also support legitimate collaboration with peer workers, and consolidate their distinct roles within the team (cf. 50).
• Ongoing measures of service users’ recovery capital (see 51) can also be used to inform strategies to build recovery capital, and, to ensure utility in clinical settings, service providers and peer workers should seek to become aware of local services and community resources.

### 4.1 Limitations of this review

Notwithstanding the strengths of this review, some of its limitations should be addressed. First, the exclusive inclusion of English-language studies may have eliminated important findings. Second, with its focus on published scientific articles, there is a risk of publication bias. Another potential source of bias is that a review protocol was not developed beforehand. Lastly, and in keeping with the indications for a scoping review, we focused on understanding the potential scope of the available literature rather than assessing the quality of studies (52). A high priority for future research is to explore the system-level barriers that may impede professionals from developing activities in a recovery orientation and to understand how care systems could better support recovery-oriented care. Furthermore, given the emphasis on the relationship between the service user and provider, and the known stigma that has been directed at people with substance use disorders, exploring the recovery orientation of care for various sub-groups of people with SUD (e.g., prisoners or offenders with mental health disorders deemed not criminally responsible), is an important avenue for further inquiry.

## 5 Conclusions

Taken together, these studies demonstrate a growing interest in recovery-supportive interventions in the scholarly literature. To advance the field, more context-specific studies are required on supporting peer professionals, (including enabling cooperation with service users, and hiring experts by experience as staff), and training of professionals (e.g., nurses, psychologists, social workers, physicians) in the principles of recovery and their practical application. However, even when professionals are well-traineds and committed to the tenets of recovery, the treatment system’s structure and policies must also support the effective implementation of recovery-supportive interventions. The extent to which these real-world and context-specific aspects are incorporated will be crucial for the design and further uptake of these interventions. The ambition of this review was to stimulate further interest in the topic.

## Data availability statement

The original contributions presented in the study are included in the article/**Supplementary Material**. Further inquiries can be directed to the corresponding author.

## Author contributions

DS: Conceptualization, Data curation, Methodology, Writing – original draft, Writing – review & editing. MC: Conceptualization, Writing – review & editing. CR: Conceptualization, Writing – review & editing. JM: Conceptualization, Writing – review & editing. PN: Conceptualization, Supervision, Writing – review & editing. WV: Conceptualization, Supervision, Writing – review & editing.
