# Supplementary material for: Recovery-supportive interventions for people with substance use disorders: a scoping review
Source: Front Psychiatry. 2024 Mar 21;15:1352818. doi: 10.3389/fpsyt.2024.1352818 (PMC10991812; doi:10.3389/fpsyt.2024.1352818)
Supplement: Supplementary file 1 [file DataSheet_1.pdf]

frontiers

Supplementary Material

Table S1. Exact search algorithms for the three databases.

Databases	Where was the search done?	
Web of Science	Topic (abstract, title, keywords)	Search 1<br>**"recovery-oriented intervention"** (Topic) or **"recovery-oriented approach"** (All Fields) or **"recovery-oriented practice"** (All Fields) or **"recovery-oriented care"** (All Fields) or **"recovery-oriented service"** (All Fields) or **"recovery-oriented model"** (All Fields) or **"recovery-supportive"** (All Fields)<br><br>Search 2<br>**"substance use"** (Topic) or **"substance misuse"** (All Fields) or **"substance abuse"** (All Fields) or **"substance dependence"** (All Fields) or **"substance use disorder"** (All Fields)<br>Combined and then filtered for Timespan: 2000-01-01 to 2023-07-31 (Publication Date) and English-language
PubMed	Title, abstract	Search 1<br>((((((("recovery-oriented intervention") OR ("recovery-oriented approach")) OR ("recovery-oriented practice")) OR ("recovery-oriented care")) OR ("recovery-oriented service")) OR ("recovery-oriented model")) OR ("recovery-supportive")<br>"recovery-oriented intervention"[All Fields] OR "recovery-oriented approach"[All Fields] OR "recovery-oriented practice"[All Fields] OR "recovery-oriented care"[All Fields] OR "recovery-oriented service"[All Fields] OR "recovery-oriented model"[All Fields] OR "recovery-supportive"[All Fields]<br><br>Search 2<br>(((("substance use") OR ("substance misuse")) OR ("substance abuse")) OR ("substance dependence")) OR ("substance use disorder")<br>"substance use"[All Fields] OR "substance misuse"[All Fields] OR "substance abuse"[All Fields] OR "substance dependence"[All Fields] OR "substance use disorder"[All Fields]<br><br>Combined Search<br>((((((("recovery-oriented intervention") OR ("recovery-oriented approach")) OR ("recovery-oriented practice")) OR ("recovery-oriented care")) OR ("recovery-oriented service")) OR ("recovery-oriented model")) OR ("recovery-supportive")) AND ((((("substance use") OR ("substance

		misuse")) OR ("substance abuse")) OR ("substance dependence")) OR ("substance use disorder"))<br>((((((("recovery-oriented intervention") OR ("recovery-oriented approach")) OR ("recovery-oriented practice")) OR ("recovery-oriented care")) OR ("recovery-oriented service")) OR ("recovery-oriented model")) OR ("recovery-supportive")) AND ((((("substance use") OR ("substance misuse")) OR ("substance abuse")) OR ("substance dependence")) OR ("substance use disorder")) Filters: English, from 2000 – 2023
Scopus	Abstract, title, keywords	Search 1<br><br>(TITLE-ABS-KEY ({recovery-oriented intervention}) OR TITLE-ABS-KEY ({recovery-oriented approach}) OR TITLE-ABS-KEY ({recovery-oriented practice}) OR TITLE-ABS-KEY ({recovery-oriented care}) OR TITLE-ABS-KEY ({recovery-oriented service}) OR TITLE-ABS-KEY ({recovery-oriented model}) OR TITLE-ABS-KEY ({recovery-supportive}))<br><br>Search 2<br>(TITLE-ABS-KEY ({substance use}) OR TITLE-ABS-KEY ({substance misuse}) OR TITLE-ABS-KEY ({substance abuse}) OR TITLE-ABS-KEY ({substance dependence}) OR TITLE-ABS-KEY ({substance use disorder}))<br><br>**1.1 ((TITLE-ABS-KEY (*{recovery-oriented intervention}*) OR TITLE-ABS-KEY (*{recovery-oriented approach}*) OR TITLE-ABS-KEY (*{recovery-oriented practice}*) OR TITLE-ABS-KEY (*{recovery-oriented care}*) OR TITLE-ABS-KEY (*{recovery-oriented service}*) OR TITLE-ABS-KEY (*{recovery-oriented model}*) OR TITLE-ABS-KEY (*{recovery-supportive}*))) AND ((TITLE-ABS-KEY (*{substance use}*) OR TITLE-ABS-KEY (*{substance misuse}*) OR TITLE-ABS-KEY (*{substance abuse}*) OR TITLE-ABS-KEY (*{substance dependence}*) OR TITLE-ABS-KEY (*{substance use disorder}*))) AND PUBYEAR > *1999* AND PUBYEAR < *2024* AND (LIMIT-TO (LANGUAGE , *"English"*))**
